# Exercise Induced Adipokine Changes and the Metabolic Syndrome

**DOI:** 10.1155/2014/726861

**Published:** 2014-01-19

**Authors:** Saeid Golbidi, Ismail Laher

**Affiliations:** Department of Pharmacology and Therapeutics, Faculty of Medicine, University of British Columbia, Vancouver, BC, Canada V6T 1Z3

## Abstract

The lack of adequate physical activity and obesity created a worldwide pandemic. Obesity is characterized by the deposition of adipose tissue in various parts of the body; it is now evident that adipose tissue also acts as an endocrine organ capable of secreting many cytokines that are though to be involved in the pathophysiology of obesity, insulin resistance, and metabolic syndrome. Adipokines, or adipose tissue-derived proteins, play a pivotal role in this scenario. Increased secretion of proinflammatory adipokines leads to a chronic inflammatory state that is accompanied by insulin resistance and glucose intolerance. Lifestyle change in terms of increased physical activity and exercise is the best nonpharmacological treatment for obesity since these can reduce insulin resistance, counteract the inflammatory state, and improve the lipid profile. There is growing evidence that exercise exerts its beneficial effects partly through alterations in the adipokine profile; that is, exercise increases secretion of anti-inflammatory adipokines and reduces proinflammatory cytokines. In this paper we briefly describe the pathophysiologic role of four important adipokines (adiponectin, leptin, TNF-***α***, and IL-6) in the metabolic syndrome and review some of the clinical trials that monitored these adipokines as a clinical outcome before and after exercise.

## 1. Introduction

The metabolic syndrome refers to a group of symptoms including obesity, high blood pressure, insulin resistance, and hyperlipidemia, in which the role of central obesity is critical [1, 2]. The increased prevalence of obesity in both industrialized and developing countries is associated with a surge in the preponderance of metabolic syndrome. In North America, 55% of 97 million adults are either overweight or obese (with a body mass index (BMI) ≥25) [3]. In a study of 12363 US men and women using the National Cholesterol Education Program Adult Treatment Panel III guidelines, the metabolic syndrome was diagnosed in 22.8% and 22.6% of the men and women, respectively [4]. The metabolic syndrome can be present in different forms, according to the combination of the various components of the syndrome, and it is well established that the metabolic syndrome increases the risk for the development of cardiovascular disease, type II diabetes, and cancer [5–7]. It is not yet known how the metabolic syndrome is triggered or how the different components are causally linked, but insulin resistance is strongly suspected as a common pathophysiologic link [8, 9]. It is clear that there is a positive correlation between body weight and insulin resistance; moreover, the risk of developing all the metabolic abnormalities is strongly associated with insulin resistance [9]. Dramatic increases in the prevalence of obesity during the second half of the last century have squarely placed adipose tissue at the center of scientific interest. This tissue now is not considered only as passive reservoir for storing excess energy substrates. Instead, adipose tissue is currently regarded as a highly metabolically active tissue that secretes many cytokines. Adipose tissue-derived cytokines or adipokines are involved in regulation of many vital processes such as energy metabolism, inflammation, and atherosclerosis. Thus, increased levels of adipokines and proinflammatory cytokines, such as TNF-*α*, have prominent roles in the pathogenesis of the metabolic syndrome. Many studies confirm that the presence of the metabolic syndrome or any of its components correlates with the levels of adipokines [10]. Several studies have established an inverse relationship between the amount of physical activity and proinflammatory cytokines in obesity, diabetes, and the metabolic syndrome. Many believe that the beneficial effect of exercise is partly mediated through changes in the adipokines profile, that is, by increasing anti-inflammatory cytokines and decreasing proinflammatory ones [11–13]. This effect has been described at the levels of gene expression, protein ligands, and receptor bindings [14]. For instance, exercise increases insulin sensitivity through reduction of resting levels of TNF-*α* and CRP and augmentation of adiponectin levels [15]. This review summarizes some of the recent findings on the role of adipokines in obesity and the metabolic syndrome and how exercise may affect these changes. Unfortunately, there are not enough data available for most of the adipokines; thus, we selected adiponectin, leptin, TNF-*α*, and IL-6 as there is a reasonable amount of data on exercise induced changes on the profile of these adipokines.

## 2. Physiologic Role of Adipokines

### 2.1. Adiponectin

Human adiponectin consists of 244 amino acids and has a distinct domain structure with a collagen-like and a globular C1q-like domain (similar to the complement component C1q). This adipokine circulates in the blood in at least three homomeric complexes: trimer (low-molecular weight form, LMW), hexamer (medium molecular weight form, MMW), and higher order multimers (high molecular weight form, HMW) [16, 17]. Plasma concentrations reveal a sexual dimorphism, with females having higher levels than males [18]. The HMW form may be the most biologically active form regulating glucose homeostasis [19, 20]. Although some studies show that the HMW form has a greater association with cardiovascular diseases [21], it has similar utility for the identification of insulin resistance and metabolic disturbances as does total adiponectin [22]. As opposed to other adipocytokines, plasma levels of adiponectin inversely correlates with body mass index (BMI), intra-abdominal fat, and indices of insulin resistance [23]. Plasma levels of adiponectin decrease with weight gain and are increased by weight loss [24, 25]. Many studies suggest that adiponectin is an important regulator of insulin sensitivity and glucose homeostasis, with several reports confirming an inverse relationship between insulin resistance and type II diabetes with plasma adiponectin levels [26–29]. It decreases hepatic glucose production and improves glucose uptake and fatty acid oxidation in skeletal muscles [30, 31]. Adiponectin stimulates insulin secretion *in vivo* [32] while hypoadiponectinemia is associated with beta cell dysfunction [33, 34]. Other studies show that adiponectin has anti-inflammatory effects, such as inhibition of endothelial nuclear factor kappa B (NF-*κ*B), suppression of phagocytic activity, and TNF-*α* production in macrophages [26, 35, 36]. It also reduces the progression of atherosclerosis by decreasing the expression of adhesion molecules, reducing proliferation of vascular smooth muscle, and blocking transformation of macrophages to foam cells [37, 38]. Crossing adiponectin transgenic mice with leptin deficient *ob/ob* or apoE-deficient mice resulted in amelioration of insulin resistance, improved beta cell degranulation, increased expression of molecules involved in fatty acid oxidation, and attenuation of atherosclerosis [39]. Likewise, adiponectin null mice display severe hepatic insulin resistance [40]. On the other hand, adiponectin administration enhances insulin effects, improves glucose metabolism [30], and increases fatty acid oxidation and weight reduction [41].

Adiponectin exerts its function through activation of two kinds of receptors, adiponectin receptor 1 (AdipoR1) and adiponectin receptor 2 (AdipoR2). AdipoR1 receptors are found in different tissues and are connected to activation of 5′ AMP-activated protein kinase (AMPK) pathways while AdipoR2 receptors are mostly expressed in the liver and mainly linked to the activation of peroxisome proliferator-activated receptor alpha (PPAR-*α*), reducing inflammation and oxidative stress [42]. Adenoviral selective expression of AdipoR1 receptors in *db/db* mice leads to activation of AMPK and decreased expression of gluconeogenic enzymes such as glucose-6-phosphatase and phosphoenolpyruvate carboxykinase 1. Increased expression of enzymes regulating glucose uptake (such as glucokinase and PPAR-*α*) results from enhanced hepatic expression of AdipoR2 receptors [42]. Expression of both receptors augments fatty acid oxidation and improves diabetes. Conversely, disruption of these receptors reduces the activity of related pathways and leads to significant glucose intolerance and aggravation of diabetes that is accompanied by increased hepatic triglyceride, inflammation, and oxidative stress [42].

The adiponectin gene is located on chromosome 3q27, which is related to type II diabetes and the metabolic syndrome [43, 44]. Several common genetic variations of the human adiponectin gene have been identified. However, a limited number of single nucleotide polymorphisms (SNPs) have been associated with obesity, type II diabetes and coronary artery disease [43, 45–47]. The accumulated evidence thus supports the idea that obesity related diseases result from an interaction between genetic and environmental causes.

### 2.2. Effect of Exercise on Adiponectin Levels

Since exercise reduces insulin resistance and facilitates glucose metabolism, several studies have attempted to establish a relationship between exercise, adiponectin levels (or the expression of adiponectin receptors), and improvements in insulin function. However, in interpreting the findings of these experiments, it is necessary to consider the intensity and duration of the exercise protocols used and the diversity of human subjects. The form of measured adiponectin (total or multimers) is another variable. Overall, it would appear that acute episodes of mild or moderate exercise in healthy, lean subjects do not affect adiponectin levels [48–50]. A decrease in adiponectin levels occurs after acute strenuous rowing by young athletes; however, longer bouts of exercise are accompanied by increased expression of adiponectin mRNA levels in skeletal muscle [51]. In case of obese individuals, Jamurtas et al. evaluated the effects of a submaximal aerobic exercise bout on adiponectin, resistin, and insulin sensitivity in nine healthy overweight males. They found no significant correlation between assessed variables except among insulin level and insulin sensitivity (decreased postexercise insulin levels and increases in insulin sensitivity) [52]. Numao et al. investigated the influence of acute exercise of various intensities on changes in the concentrations of total adiponectin and adiponectin oligomers (HMW versus combination of LMW and MMW) in nine middle-aged abdominally obese men [53]. High intensity exercise decreased total adiponectin concentrations mainly by reducing LMW and MMW adiponectin levels without changing HMW adiponectin [53]. Conversely, a recent study of plasma adiponectin levels in inactive, abdominally obese men showed that both acute and short term (one week) aerobic exercise training significantly increased plasma values [54]. Chronic exercise protocols in both healthy and insulin resistant subjects also produced conflicting results in the literature, as shown in Table 1, which summarizes the results of some exercise protocols on adiponectin levels.

### 2.3. Leptin

The hormone leptin, whose nomenclature is derived from the Greek word “leptos” (means thin), is a 16 kDa protein that has a primary role in suppressing appetite and increasing energy expenditure through metabolism. Leptin is primarily made in adipose tissue and its circulating levels correlate with body fat stores [55]. It is also expressed in the placenta, ovaries, mammary epithelium, bone marrow, and lymphoid tissues [56, 57]. In humans, the leptin gene is located on chromosome 7 [58]. So far, six types of receptors have been recognized for leptin (Ob-Ra-Ob-Rf) that are all encoded by a single gene (LEPR). Ob-Re does not encode a transmembrane domain and is secreted and circulates in human plasma and represents the primary leptin-binding activity [59]. Ob-Ra and Ob-Rc have significant roles in transporting leptin across the blood brain barrier [60]. Ob-Rb is the only receptor isoform that signals by intracellular mechanisms and this receptor is scattered throughout the central nervous system (CNS), particularly in hypothalamus, where it regulates energy homeostasis and neuroendocrine function [61, 62]. Obesity and metabolic derangement in *db/db* mice are the consequences of dysfunctional Ob-Rb receptors. Janus-activated kinase (JAK), signal transducers and activators of transcription (STAT), insulin receptor substrate, and the mitogen-activated protein kinase (MAPK) pathways are important leptin intracellular signaling mechanisms [63]. The binding of leptin to its receptor leads to the formation of the Ob-R/JAK2 complex and activation of STAT3, which is phosphorylated and migrates to the nucleus presumably to affect changes in gene expression [64]. Binding of leptin receptors to JAK2 also results in JAK2 autophosphorylation [65], which in turn phosphorylates insulin receptor substrate proteins and involvement of phosphatidyl inositol 3-kinase to activate downstream signals [66].

Leptin is one of the best-known hormone markers for obesity and is very sensitive to levels of energy intake, particularly in energy deficient states. Two or three days of fasting lowers human plasma leptin levels even before any loss in body fat mass occurs [67, 68]. Decreased leptin levels set off a series of biological reactions, including a reduction of sympathetic nervous system activity, thyroid hormones, hypothalamic gonadotropin-releasing hormones, insulin-like growth factor I (IGF-I), and augmentation of growth hormone (GH) and adrenocorticotropic hormone (ACTH), to reduce energy expenditure and prevent weight loss [69–73]. Conversely, adequate leptin levels promote energy expenditure through different effects on the endocrine (e.g., growth, reproduction, and immune system) and autonomic nervous system. Leptin deficiency in animals (*ob/ob* mice) and humans results in increased food intake, decreased energy expenditure, and infertility [74]. In spite of the appetite-lowering effects of leptin, the majority of obese individuals (except for rare cases of congenital leptin deficiency) show hyperleptinemia. These people are thought to be leptin resistant. The precise mechanisms of leptin unresponsiveness in obese individuals are yet to be determined; however, several mechanisms have been proposed to explain this phenomenon. Using an animal study, El-Haschimi et al. suggested that leptin may be unable to reach sites of action in the hypothalamus and/or that an intracellular reduction of leptin-mediated STAT signaling occurs [75]. Increased expression of suppressor of cytokine signaling-3 (SOCS-3), an inhibitor of postreceptor leptin signaling that lessens most of the ObRb signaling at chronically high levels of circulating leptin, is proposed as another mechanism [76]. Increased expression of SOCS-3 occurs in the vastus lateralis muscle of obese individuals [77] and diet-induced obese animals [78]. Similarly, SOCS3 deficient mice are protected against the development of hyperinsulinemia and insulin resistance during high fat diet induced obesity. These animals have increased expression of skeletal muscle insulin receptor substrate-1 (IRS-1) and Akt phosphorylation that results in increased skeletal muscle glucose uptake [79]. Persistent chronic inflammation and increased level of TNF-*α* may also play a role in hyperleptinemia of obese individuals as a positive correlation has been shown between TNF-*α* and leptin levels in both human and rodents [80, 81]. The regulatory role of insulin and the effect of chronic hyperinsulinemia in increased leptin levels are another mechanism which remains to be clarified. In rodent, the stimulatory effect of insulin on leptin expression and secretion has been shown [82, 83]; however, such studies in human being were inconclusive [84].

### 2.4. Effect of Exercise on Leptin Levels

Because of the multifaceted role of leptin in human metabolism, many investigators evaluated the effect of different exercise protocols on leptin levels. Acute and short-term bouts of exercise do not affect leptin levels in healthy individuals [85]. However, longer durations of exercise (≥60 min) that are associated with increased energy expenditure (≥800 kcal) can decrease leptin concentrations [86]. In an experiment with 45 men who participated in one of three competitive exercise protocols with approximately 1400, 5000, and 7000 Kcal energy expenditure, only the participants in the last two categories had reduced serum leptin levels; prompting the authors to conclude that only prolonged endurance exercise with large energy expenditure reduces circulating serum leptin levels [87]. Short-term exercise training (≤12 weeks) is not associated with significant changes in leptin levels, yet there are variable reports when training courses last more than 3 months. Generally speaking, those training protocols which lower adiposity will result in diminished leptin levels.

An important point of interest in measuring leptin levels is paying attention to diurnal variations in its blood levels. Kraemer et al. determined leptin levels in 15 healthy postmenopausal women at baseline, exercise, and recovery point intervals. Blood sampling with the same time intervals but without exercise was performed one month later as a control group. Even though no difference was detected between two groups, there was a gradual decrease from baseline levels to postexercise and recovery period. They emphasized the need to account for diurnal variations in measuring leptin levels over the course of exercise trials [88].

Diabetic patients seem to be more responsive to the leptin lowering effects of exercise, as acute and short bouts of exercise can reduce leptin levels in such patients. Kanaley et al. reported a decrease in leptin levels after an acute episode of exercise in diabetic (not healthy) subjects, as measured after 6 weeks of training [89]. This increased sensitivity to exercise-induced lowering effect of plasma leptin levels remains in the offspring of diabetic patients [90].  Table 2 summarizes some of the clinical trials which have studied the effects of exercise on leptin levels in different groups of people.

### 2.5. Tumor Necrosis Factor-Alpha (TNF-*α*)

TNF-*α* is a cytokine that is mainly produced by monocytes and macrophages. It is also secreted by other immunogenic cells such as CD4 lymphocytes and natural killer cells and plays major roles in cell death (apoptosis), inflammation, and induction of acute phase reactants. In obese individuals, macrophage-infiltrated visceral fat is the main site of TNF-*α* production [91]. Expression levels of the TNF gene are higher in abdominal adipose tissue compared to subcutaneous fat, and, importantly, greater TNF gene expression occurs in the adipose tissues of obese animals [92] and humans [93]. Accumulating data suggests a direct relationship between TNF-*α* plasma levels and insulin resistance. For instance, in both *ob/ob *and diet-induced obese mice, genetic deletion of TNF-*α* or its receptors significantly reduced insulin resistance and improved insulin signaling in muscle and adipose tissue [94]. Also, based on a community-based cohort study, it is proposed that the prevalence of insulin resistance increases with greater levels of resistin and TNF-*α* and is inversely related to adiponectin levels [95]. Diabetic patients have high activity of TNF-*α* in the plasma and skeletal muscles [96–98].

**Figure 1:**
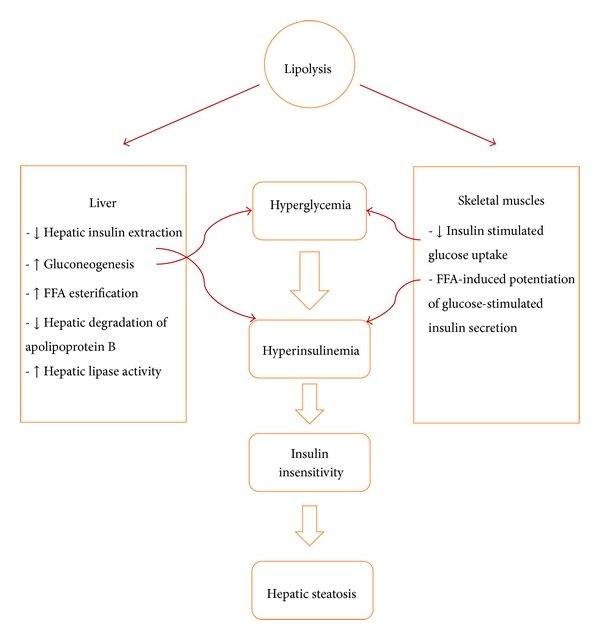
Some of the pathophysiologic mechanisms involved in the pathogenesis of fatty liver.

At a cellular level, TNF-dependent activation of stress-related kinases inhibits insulin signaling, causing cellular insulin resistance. Some of these stress-related kinases also promote further production of TNF, perpetuating a positive feedback mechanism for sustained TNF activity and chronic insulin resistance [99]. Targeted disruptions of genes encoding TNF [94] or TNF receptors [100] markedly improve insulin sensitivity in obese mice. On the other hand, visceral fat obesity is associated with decreased concentrations of insulin-sensitizing and anti-inflammatory adipokines [101]. During lipolytic activity, more fatty acids are released from visceral adipose tissue compared to subcutaneous adipose tissue [102, 103], as visceral fat has a higher metabolic rate and has increased susceptibility to lipolytic enzymes [104]. Antilipolytic activity of insulin also has a lesser influence on visceral fat [105] (Table 3). Increased TNF level induces hepatic uptake of these fatty acids in a process that is accompanied by reduced fatty acid oxidation and triglyceride export. These events cause accumulation of fat within hepatocytes (hepatic steatosis). Direct drainage of visceral fat-induced FFAs through porta vein is another factor in the pathogenesis of fatty liver. Indeed, nonalcoholic fatty liver disease commonly accompanies the metabolic syndrome. It is generally believed that the chain of reactions leading to hepatocyte fatty degeneration begins with increased levels of TNF and insulin resistance, which precede fat accumulation [106]. During hepatic insulin resistance, hepatic glucose production is no longer downregulated by insulin, resulting in increased hepatic glucose production and stimulation of increased insulin secretion. Chronic hyperinsulinemia desensitizes peripheral tissues to insulin and causes systemic insulin resistance. Insulin resistance increases adipocyte lipolysis, resulting in the release of large amounts of fatty acids into the blood and exacerbation of hepatic steatosis and insulin resistance [107] (Figure 1). TNF-*α* also promotes the incorporation of fatty acids into diacylglycerol, which may contribute to the development of TNF-*α* induced insulin resistance in skeletal muscle [108].

TNF is also a potent inducer of mitochondrial ROS and increases ROS production in fatty hepatocytes [109]. In order to mitigate or reverse this chronic oxidative stress, adaptive mechanisms such as uncoupling proteins are activated or upregulated. The controlled transfer of protons can uncouple mitochondrial respiration across the inner mitochondrial membrane, thereby dissipating the proton gradient and reducing the harmful effects of ROS. The inner mitochondrial membrane uncoupling proteins play important roles in thermogenesis of brown adipose tissue and in regulating the disposal of mitochondrial ROS in other tissues [110]. Decreases in the mitochondrial membrane potential reduce ATP synthesis and make cells susceptible to necrotic cell death [111]. These events lead to local inflammatory reactions by attracting inflammatory cells, leading to the histopathology of nonalcoholic steatohepatitis [112].

### 2.6. Effect of Exercise on TNF-*α* Level

A large body of evidence shows an inverse relationship between plasma levels of inflammatory adipokines and the amount of physical activity. Even though acute episodes of exercise might be associated with increased levels of inflammatory cytokines, exercise training reduces circulating levels of inflammatory markers, even in lean individuals [113]. Controlling the release and activity of at least two cytokines, namely, TNF-*α* and IL-6, could contribute to the natural protective effects of physical activity in the metabolic syndrome. Exercise confers protection against TNF-*α* induced insulin resistance [114] while it reduces CRP, IL-6, and TNF-*α* levels and increases anti-inflammatory substances such as IL-4 and IL-10 [115, 116]. The association between the metabolic syndrome and inflammation is well documented [117, 118]. The reduction in TNF-*α* by exercise may be exerted through both IL-6 (muscle-derived) dependent and independent pathways [119, 120]. Furthermore, exercise induced increases in epinephrine levels can also blunt the TNF-*α* response by a poorly defined mechanism [121]. Weight reduction through exercise (and diet) decreases the volume and number of adipocytes and also reduces the number of endothelial and macrophage cells that are lodged inside adipose tissue that produce proinflammatory mediators. Increased production of anti-inflammatory mediators by adipocytes and decreased hepatic production of fibrinogen and other proinflammatory mediators are other consequences of exercise-induced weight reduction. Weight loss also influences the immune system by reducing the number of mononuclear cells in the circulation; these are important sources of proinflammatory cytokines [122]. The effect of exercise training on reducing the expression of TNF-*α* in white adipose tissue has been shown in several animal studies, as well [123–125].  Table 4 summarizes the results of some clinical trials that measured TNF-*α* after exercise training in humans.

### 2.7. Interleukin-6 (IL-6)

IL-6 belongs to a family of cytokines that collectively have an important role in immune reactions, hematopoiesis, and metabolism. IL-6 has both pro- and anti-inflammatory effects and is classified as an adipokine as well as a myokine and causes a wide range of sometimes contradictory effects. The physiologic nature of target cells and specific *in vivo *conditions are confounding factors in determining its final biological effect [126]. Indeed, IL-6 is a good example of a chemical that is able to cause cross talk amongst different tissues.

There are conflicting reports about the effect(s) of IL-6 on lipid and glucose metabolism and insulin sensitivity. The release of the adipokine IL-6 is related to BMI [127–129]. Fernandez-Real et al. demonstrated a positive association between IL-6 concentrations and the fasting insulin resistance index in 228 healthy volunteers [130]. Subcutaneous injections of recombinant human IL-6 also increase blood glucose and glucagon levels without changes in C-peptide levels, supporting the idea that IL-6 alters insulin sensitivity [131]. Moreover, IL-6 impairs insulin signaling in adipocytes and reduces insulin-dependent glucose uptake by reducing GLUT4 expression and IRS-1 [132]. The disturbing effect of IL-6 on insulin signaling also occurs in mouse hepatocytes and human hepatocarcinoma cells [133]. These data collectively suggest that IL-6 impairs insulin sensitivity. On the other hand, Carey et al. reported that infusion of IL-6 to seven healthy males accelerated glucose removal with no effects on endogenous glucose production during a hyperinsulinemic-euglycemic clamp study. They also reported that IL-6 improved basal and insulin-stimulated glucose uptake by myocytes, an effect that was mediated by translocation of GLUT4 transporters to the plasma membrane [134]. Similarly, an acute infusion of IL-6 improved insulin sensitivity and glucose removal in animal studies [135]. One explanation for this discrepancy in the effects of IL-6 effect on glucose metabolism could be the time course of IL-6 elevation. Chronic elevation of IL-6 levels in obese and type II diabetics may be associated with insulin resistance, while acute transient increase can enhance insulin sensitivity. However, this speculation is not supported in an animal study in which chronic infusion (14 days) of IL-6 increased insulin sensitivity [135].

IL-6 mRNA is upregulated in contracting skeletal muscle [136], and the transcriptional rate of the IL-6 gene is also markedly enhanced by exercise [137]. Physical training augments the expression of IL-6 receptors in human skeletal muscle and sensitizes them to IL-6 at rest [138]. Of interest is that connective tissue located in and around working muscles may have an additional role in the production and secretion of IL-6 to the plasma [139]. Availability of energy resources can also affect intramuscular IL-6 mRNA levels in response to exercise as this response is higher under glycogen depleted conditions [140] and following prolonged and strenuous activities [141]. Lipid turnover, lipolysis, and fat oxidation, via activation of AMP-activated protein kinase are enhanced by IL-6 [142]. This is supported by studies showing that IL-6 deficient mice (IL-6^−^/^−^) develop mature onset obesity and have disturbed carbohydrate and lipid metabolism that is partly reversed by IL-6 replacement [143]. Infusion of IL-6 to human subjects increases systemic fatty acid oxidation [144]. The lipolytic effect of IL-6 on fat metabolism was confirmed in two clinical studies of healthy and diabetic subjects [142, 145]. A recent study reported that exercise-induced increases in IL-6 production could improve insulin secretion through stimulating glucagon-like peptide-1 (GLP-1) secretion, a hormone that induces insulin secretion. This newly explained mechanism adds to the importance of IL-6 as a mediator of cross talk between different tissues [146].

Collectively, it can be inferred that IL-6 has regulatory effects on metabolism of both adipose and muscular tissue and is a mediator of cross talk between these two compartments. Excessive production of IL-6, as an adipokine, in obesity and diabetes, has an adverse effect on glucose metabolism and insulin sensitivity. On the other hand, as a muscle-secreted IL-6 myokine, it enhances glucose disposal and lipolysis and mediates the beneficial effects of physical activity. Another theory for explaining the increased amounts of IL-6 in obesity and insulin resistance states is that elevated levels of IL-6 are a secondary defense response to higher amounts of TNF-*α*. In other words, TNF-*α*, as an important pathophysiological culprit in obesity, stimulates IL-6 release. Alternatively, increased amounts of IL-6 may represent a compensatory mechanism in insulin resistance conditions for maintaining glucose homeostasis. Furthermore, increased levels of IL-6 may be a response to impaired IL-6 signaling [147], which is poorly defined in many clinical studies of obesity, insulin resistance, and diabetes.

### 2.8. Effect of Exercise on IL-6 Level

Interleukin-6 (IL-6) is the first cytokine to be released into the circulation during exercise, and its levels increase in an exponential fashion in response to physical exertion [148]. Exercise-induced increases in plasma IL-6 correlate with the muscle mass involved in exercise activity and also with the mode, duration, and, especially, the intensity of exercise [149]. The infusion of recombinant human IL-6 (rhIL-6) into human subjects simulates the exercise induced IL-6 response in the prevention of endotoxin-induced increase in plasma TNF-*α* [150]. IL-6 inhibition of LPS-induced TNF-*α* production has also been shown in cultured human monocytes and IL-6 deficient knockout mice [151, 152]. Furthermore, IL-6 stimulates the release of other anti-inflammatory cytokines including IL-10 and IL-1Ra [153]. These and other experiments suggest that the anti-inflammatory effects of exercise are partly mediated through increased levels of IL-6.

Focusing on the results of clinical trials that have measured IL-6 reveals the following generalities. First, the magnitude of IL-6 increment is higher after moderate to severe exercise in untrained individuals. Exercise training decreases the magnitude of IL-6 response following strenuous activity. Second, exercise decreases blood levels of IL-6 in the metabolic syndrome and obese patients, while normal weight individuals experience increased levels of IL-6 when it is released as a myokine. Genetic polymorphism is another confounding factor which might explain differences in exercise-induced IL-6 variations [154].  Table 5 summarizes the results of some experiments in which IL-6 was part of laboratory outcomes in clinical studies.

## 3. Conclusion

**Figure 2:**
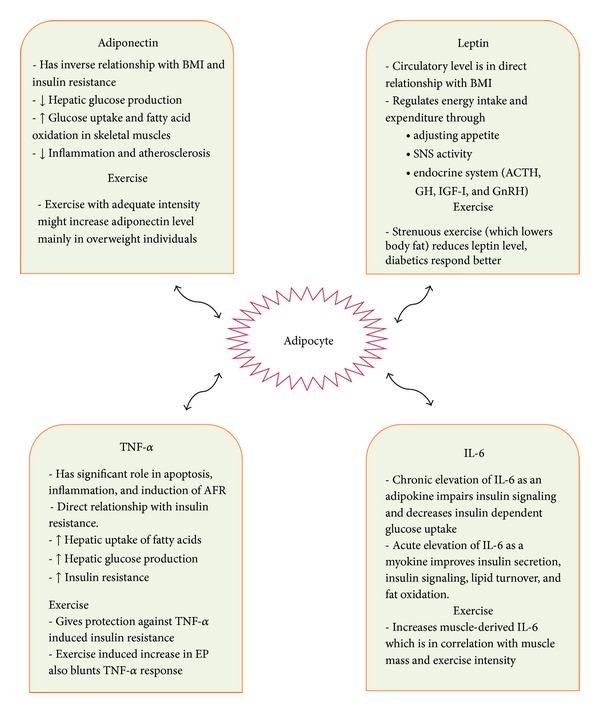
Selected physiologic effects of four adipokines and the effect of exercise on their blood levels.

In reviewing the information on the effects of exercise on adipokine levels (Figure 2), several drawbacks hinder lining a general conclusion. First, there is lack of unified standards in measuring the exercise intensity. These standards could be different among patients with various BMI levels as obese individuals may benefit more than normal-weight people from a certain level of physical activity. Exercise should exceed a defined level that is known to bring about physiologic changes. These thresholds have not been defined precisely in different groups of people. The *in vivo* interaction between different adipokines is another point which has mostly been ignored in isolated cytokines measurements. For instance, higher levels of adiponectin hinder the secretion of TNF-*α* and IL-6 [155], while TNF-*α* negatively affects adiponectin production and enhances IL-6 production [78]. There are also many other adipokines which yet have not been investigated in this context. Unraveling the complex physiology and relations between various adipokines can lead to a better understanding of sport physiology.

## Figures and Tables

**Table 1:** Summary of selected clinical trials in which adiponectin levels have been measured as a clinical outcome before and after exercise.

Number	Subjects	Exercise intensity	Measured parameters	Results
Measurement of adiponectin after exercise in healthy subjects				
48	Eight male and 8 female healthy subjects	60 min stationary cycle ergometry session at 65% VO_2_ max	Plasma adiponectin, TNF-*α*, insulin, glucose, and leptin	Neither male nor female subjects showed changes in adiponectin and leptin concentrations
156	Six healthy male	(i) First experiment: 30 min of heavy continuous running at 79% of VO_2_ max (ii) Second experiment: strenuous intermittent exercise consisting of treadmill running at 60, 75, 90, and 100% of VO_2_ max	Serum concentration of adiponectin, insulin, and plasma concentrations of glucose and lactate	No significant change in adiponectin concentrations in either of these protocols
49	Ten active male subjects	Two similar trials, each trial consisted of 120 min cycling exercise at 50% VO_2_ max	(i) Plasma adiponectin, FFA, and glycerol concentrations (ii) Determining adiponectin protein and adiponectin receptor mRNA expression in skeletal muscle	No change in plasma adiponectin concentration and tissue mRNA expression of adiponectin receptors
157	24 healthy male	Endurance or resistance training 3 days/week for 12 weeks (i) Endurance training (continuous running at 75–85% of MHR) (ii) Resistance training (4 sets of circuit weight training for 11 stations at 50–60% of one-repetition maximum)	Serum glucose, insulin, and adiponectin	Endurance and resistance training were not accompanied by increased adiponectin levels

Measurement of adiponectin after exercise in obese subjects				
158	16 obese men and women (age, 63)	12-week supervised exercise (5 days/wk, 60 min/day, treadmill/cycle ergometry at 85% of max HR)	Insulin resistance, fat mass, adiponectin, TNF-*α*, and leptin levels	Exercise reversed insulin resistance but there was no change in leptin and adiponectin levels
159	19 overweight and obese girls	12 weeks of supervised training (3 d/wk, 40 min each session)	Insulin sensitivity, adiponectin, CRP, IL-6, IGF-1, blood lipids, and so forth	Insulin sensitivity improved without change in adiponectin and leptin
160	25 obese sedentary premenopausal women	12 weeks of aerobic exercise program (5 d/wk, 50% of VO_2_ max)	Plasma and mRNA levels of leptin, adiponectin, IL-6, and TNF-*α*	Plasma leptin level decreased while plasma levels of other cytokines remained unchanged
161	26 overweight males	10 weeks of aerobic exercise (4-5 d/wk, 40 min each session, brisk walking mixed with light jogging, 55–70% VO_2_ max)	Insulin sensitivity, indirect calorimetry, and plasma adiponectin level	Adiponectin levels rose by 260% after 2-3 bouts of exercise (1 week) without any change in BW
162	Eight obese female students and 8 obese controls	7 mo exercise training (30–60 min/d, 4-5 d/wk 60–70% of HR-reserve)	Adiponectin, leptin, hs-CRP, TNF-*α*, and lipid profile	Exercise decreased BW, body fat mass, hs-CRP, leptin, and TNF-*α* and increased HDL, VO_2_ max, and adiponectin

BW: body weight; FFA: free fatty acids; HR: heart rate; hs-CRP: high sensitivity C reactive protein; MHR: maximal heart rate; VO_2_ max: maximal oxygen consumption; wk: week.

**Table 2:** Summary of selected clinical trials in which leptin levels were measured as a clinical outcome before and after exercise.

Number	Subjects	Exercise intensity	Measured parameters	Results
Acute and short (≤60 min) exercise protocols without significant effect on leptin levels				
163	Seven young men (age, 27)	30 min Ex at 25% and 75% of the difference between the lactate threshold (LT) and rest (0.25 LT, 0.75 LT), at LT, and at 25% and 75% of the difference between LT and VO_2_ peak (1.25 LT, 1.75 LT)	Leptin AUC for all six conditions	30 min Ex at different intensities does not affect leptin levels during or up to 3.5 hours after exercise
164	Six healthy untrained men	Three sessions of control, Max Ex, and prolonged Ex at 50% of VO_2_ max	Serum leptin, insulin, glucose, FFA, and glycerol REE and BF were also assessed	(i) No significant differences were observed in leptin concentrations between the control and exercise session (ii) Control serum leptin was positively correlated to BF and glucose and negatively correlated to REE
83	15 healthy postmenopausal women (8 on HRT and 7 on NHRT)	30 min treadmill at 80% VO_2_ max	Leptin level before and after exercise session and one month later as (without exercise) control values	No significant differences were observed between groups
165	Eight young, lean, sedentary men	41 min of cycle ergometry at 85% of VO_2_ max and 1-2 weeks later the same protocol but without exercise to be considered as control	Serum leptin, insulin, protein, and cortisol levels plus plasma glucose, EP, and NE concentrations	No significant changes in leptin levels
166	Ten young lean men (age, 23)	Acute effects of 3 resistance exercise protocols including MS, MH, and SE on serum leptin	Serum leptin, cortisol, glucose, and GH	Typical resistance exercise protocols did not result in serum leptin changes

Longer duration (≥60 min) exercise protocols which resulted in decreased leptin levels				
167	12 fasted men (age, 30) to work on stationary cycle ergometer and 14 nonfasted marathon runner (age, 41)	Intense exercise in both groups (four half-hour period at 75% VO_2_ max for cyclists and 101 mile running for runners)	Blood leptin levels before, at the end, 6, 18, and 24 hours after exercise	Negative energy balance of exercise can reduce serum leptin concentrations
168	29 male marathon runner compared with 22 age-, sex-, and BMI-matched sedentary controls	Marathon run (42.195 km) with a calculated energy expenditure of over 2800 Cal	Leptin levels one day before and after run	There was a significant reduction in blood leptin levels in runners
169	9 trained men (age, 22–33)	(i) A MAX short duration run (ii) 60 min endurance ran at 70% of VO_2_ max consumption (END)	Plasma leptin, insulin, and glucose levels before, immediately after, 24, and 48 hours after exercise	(i) Plasma leptin levels did not differ between time points for the MAX run (ii) Leptin was significantly lower 48 h after exercise in END group
170	Ten young men (age, 21)	Acute heavy resistance protocol (50 total set comprised of the squat, bench press, and lat pull-down)	Plasma leptin levels	Leptin concentration showed a delayed (approximately 9 h) reduction after acute resistance exercise
82	45 males participated in one of the three competitive exercise protocols	(i) A half marathon run (21.097 Km, 1400 Kcal) (ii) A ski-alpinism (45 Km, 5000 Kcal) (iii) An ultramarathon race (100 Km, 7000 Kcal)	(i) Serum leptin (ii) Plasma free fatty acids	Serum leptin levels decreased significantly in ultramarathon and ski-alpinism but not in half marathon run

Exercise protocols in obese, prediabetic, and/or metabolic syndrome patients				
171	Fifty inactive men (age, 65–78, BMI, 28.7–30)	Low intensity (*n* = 14) Moderate intensity (*n* = 12) High intensity (*n* = 14) Control (*n* = 10) For 24 weeks	Exercise energy cost Skinfold sum Body weight VO_2_ max Resting metabolic rate Plasma leptin and adiponectin	Leptin was diminished by all treatments
172	50 sedentary type II diabetic subjects divided to a diet therapy (*n* = 23) group or an exercise plus diet therapy group	Exercise protocol consisted of walking and cycle ergometer exercise for 1 h × 5/week with the intensity of 50% of VO_2_ max	Plasma leptin levels Fasting plasma insulin, glucose, cortisol, and HbA1c Urinary 17-OHCS	Leptin significantly decreased in exercise group
84	30 men and women (17 controls and 13 type II obese diabetics, age 40–55)	Three repetition of maximal weight lifting bout 72 h after their last training bout of 6 weeks of resistance training	Serum leptin levels plus glucose and insulin	Acute exercise decreased leptin level in diabetic group
85	34 women offspring of type II diabetic patients and 36 matched female controls	Seven weeks of exercise intervention	Insulin sensitivity index, VO_2_ max, and plasma leptin level	Plasma leptin levels decreased only in the offspring of diabetic patients
173	50 diabetic men divided to exercise training or standard therapy for 2 years	Endurance and muscle strength training 4 times/week.	HbA1c, insulin, leptin, blood lipids, blood pressure,VO_2_ max, and muscle strength	VO_2_ max, muscle strength, HbA1c, and leptin improved in exercise group

AUC: area under curve; BF: body fat; EP: epinephrine; Ex: exercise; FFA: free fatty acids; GH: growth hormone; HRT: hormone replacement therapy; MAX: maximum intensity; MH: muscular hypertrophy; MS: maximum strength; NE: norepinephrine; NHRT: nonhormone replacement therapy; 17-OHCS: 17-hydroxycorticosteroid; REE: resting energy expenditure; SE: strength endurance; VO_2_ max: maximal oxygen consumption.

**Table 3:** Some differences between visceral and subcutaneous adipose tissue [105].

	Visceral fat	Subcutaneous fat
Sensitivity to catecholamine induced lipolysis	Higher	Lower
Sensitivity to insulin's antilipolytic effects	Lower	Higher
Density of glucocorticoid receptors	Higher	Lower
Androgen receptors	Higher	Lower
Leptin secretion and leptin mRNA expression	Higher	Lower
Secretion of IL-6 (as an adipokine)	Higher	Lower

**Table 4:** Summary of selected clinical trials in which TNF-*α* level has been measured as a clinical outcome before and after exercise.

Number	Subjects	Exercise intensity	Measured parameters	Results
174	67 healthy, premenopausal women and 40 age matched normal weight women	Walk for at least 1 h three times a week plus a diet contained 1300 Kcal/d and behavioral counseling	Echocardiography plus circulating levels of TNF-*α*, IL-6, IL-8, and CRP	After one year, there was a significant reduction in inflammatory markers and improvement in cardiac function
175	23 overweight and obese adults are randomized into vit D + exercise and exercise group	All participants did 12-week (3 d/wk) progressive resistance exercise at 70–80% of one repetition maximum	Stimulated TNF-*α*, circulating CRP, TNF-*α*, IL-6, and ALT	Both groups had a significant reduction in nonstimulated TNF-*α* production after 12 weeks
176	82 subjects with type II diabetes and metabolic syndrome are randomized to following groups: 20 T2D (sedentary control, A) 20 T2D (low intensity aerobic exercise, B) 20 T2D (high intensity aerobic exercise, C) 22 T2D (aerobic and resistance exercise, D)	Twice a week supervised sessions of 60 min of aerobic exercise at 70–80% VO_2_ max for group C patients and 40 min aerobic exercise at 70–80% VO_2_ max + 20 min resistance exercise at 80% of 1 repetition maximum for Group D subjects. group B received counseling to perform low intensity physical activities. These protocols continued for 12 months	HbA1c, FBS, TG, TC, HDL, hs-CRP, IL-1*β*, IL-4, IL-6, IL-10, TNF-*α*, IFN-*γ*, leptin, resistin, and adiponectin VO_2_ max	(i) Significant decrease of hs-CRP in groups C and D (ii) Leptin, resistin, and IL-6 decreased in groups C & D, while adiponectin increased (iii) IL-1*β*, TNF-*α*, and IFN-*γ* decreased in group D, whereas anti-inflammatory IL-4 & 10 levels declined
177	31 inactive subjects with metabolic syndrome are divided to (i) High-intensity aerobic interval training (AIT), (ii) strength training (ST), (iii) control group	Exercise training was carried out three times per week for 12 weeks	Serum insulin, hs-CRP, IL-18, IL-6, and TNF-*α*	Serum IL-18 was reduced after AIT TNF-*α* level was lower in AIT group compared to ST and controls No changes in serum IL-6, insulin, or hs-CRP within or between the groups
178	20 obese individuals (BMI, 32) with at least one other component of the metabolic syndrome are randomized to exercise group and diet group after 8 weeks of control period	Exercise consisted of 8 weeks of moderate cycling exercise (30 min, 3 times/wk)	Fasting glucose and insulin levels Muscle biopsy for analysis of skeletal muscle TNF-*α* and GLUT4	Both interventions reduced plasma insulin levels Only diet reduced muscle TNF-*α* but exercise did not change TNF-*α* protein expression
179	47 obese diabetic patients randomly assigned to aerobic (AT, *n* = 27) or aerobic plus resistance (ART, *n* = 20) exercise protocols	AT program was 15 min row ergometer plus 15 min bicycle ergometer at 70% of HR max for 5 d/wk ART program was AT program plus 15 min resistance training at 40–50% of HR max	Blood glucose, insulin, and lipid profile Leptin, adiponectin, resistin, TNF-*α*, MCP-1, and MMP-2	Adiponectin level increased 54% after AT while decreased by 13% after ART MMP-2, TNF-*α*, and MCP-1 levels decreased in AT while increased in ART group
180	23 obese postmenopausal women underwent resistance exercise training or social interaction intervention	3 sets, 10 exercises, 3 × per week, 8–12 repetition maximum	IL-6, leptin, CRP, TNF-*α*, adiponectin, mRNA expression of TLR4, and MC1R.	TNF-*α*, CRP, and leptin reduced in exercise group without any change in body composition

ALT: alanine aminotransferase; BMI: body mass index; CRP: C reactive protein; FBS: fasting blood sugar; GLUT4: glucose transporter 4; HDL: high density lipoprotein; IFN-*γ*: interferon-gamma; MCP-1: monocyte chemoattractant protein-1; MC1R: melanocortin 1 receptor; MMP-2: matrix metalloproteinase-2; TG: triglyceride; TC: total cholesterol; TLR4: toll-like receptor 4.

**Table 5:** Summary of selected clinical trials in which IL-6 level has been measured as a clinical outcome before and after exercise.

Number	Subjects	Exercise intensity	Measured parameters	Results
181	24 insulin resistant obese individuals	Six months of moderate intensity exercise plus hypocaloric diet	IL-6, leptin, adiponectin, resistin, TNF-*α*, hsCRP, and insulin sensitivity	Plasma leptin and IL-6 decreased. TNF-*α* tended to decrease. Adiponectin increased in diabetics
182	56 obese women and 40 age-matched normal weight	One year of increased physical activity (at least 1 h walk 3 times/wk) plus energy restricted diet	Proinflammatory cytokines including TNF-*α*, IL-6, P-selectin, VCAM-1, ICAM-1 plus glucose, and lipid profile	Proinflammatory cytokines were higher in obese individuals Weight reduction was associated with decreased levels of IL-6 and TNF-*α*
183	15 athletes participants in an ultradistance foot race	246 Km Spartathlon	IL-6, CRP, SAA, free plasma DNA, and lipid profile	IL-6 (8000 fold), CRP, SAA, and free plasma DNA levels increased at the end of this acute exercise
184	49 white obese school aged children	A combined protocol of energy restriction and increased physical activity for 3 weeks	Indexes of obesity, IL-6, leptin, estradiol, systolic and diastolic BP, and HR	All determined parameters decreased significantly during 3-week program
185	17 healthy young women (YW) and 8 postmenopausal women (PMW)	Five sets of six maximal eccentric actions of the elbow flexors	CK, IL-6, IL-10, TNF-*α*, and PGE2	For YW, IL-6 and IL-10 values increased 72 h after eccentric exercise
186	11 endurance athletes	Two experimental trials consisted of 90 min run at 75% of VO_2_ max	IL-6, free Hb, haptoglobin, hepcidin, and iron parameters	Serum iron and IL-6 significantly increased after exercise
187	60 overweight/obese diabetic patient randomized to exercise or control groups	16-week aerobic exercise training consisting of four 45–60 min sessions/week (50–60% of VO_2_ max)	Insulin resistance, plasma levels of resistin, IL-6, FBS, and lipid profile	Exercise training decreased both plasma IL-6 and IL-18

BP: blood pressure; FBS: fasting blood sugar; Hb: hemoglobin; HR: heart rate; hs-CRP: high sensitivity C reactive protein; ICAM-1: intercellular adhesion molecule-1; PGE2: prostaglandin E2; SAA: serum amyloid A; VCAM-1: vascular cell adhesion molecule.
